# Comparing perioperative outcomes of stapled versus handsewn Kono-S anastomosis after ileocolonic resection for Crohn’s disease

**DOI:** 10.1007/s10151-025-03219-y

**Published:** 2025-10-16

**Authors:** E. Benshabat, J. B. Yuval, H. Leibovitzh, A. Hirsch, G. Lahat, Y. Kariv, M. Zemel

**Affiliations:** 1 Surgical Division, Tel Aviv Sourasky Medical Center affiliated with Faculty of Medical and Health Sciences, Tel Aviv University, Tel Aviv, Israel; 2 Surgical Division, Colorectal Unit, Tel Aviv Sourasky Medical Center, Tel Aviv, Israel; 3 Department of Gastroenterology and Liver Diseases, Tel Aviv Sourasky Medical Center affiliated with Faculty of Medical and Health Sciences, Tel Aviv University, Tel Aviv, Israel; 4 Department of Surgery. Colorectal Unit, Tel Aviv Sourasky Medical Center, 6 Weizmann St., 64239 Tel Aviv, Israel; 5 Tel Aviv University, Tel Aviv, Israel

**Keywords:** Inflammatory bowel disease, Crohn’s disease, Surgery, Kono-S, Ileocolonic resection, Handsewn anastomosis, Stapled anastomosis

## Abstract

**Introduction:**

Ileocecal resection is the most common surgery in Crohn’s disease (CD). As recurrences often occur at the anastomosis it has been questioned whether surgical technique may have a role in its prevention. The Kono-S anastomosis, first described in 2011, has shown potential to reduce anastomotic recurrence while maintaining luminal width and preventing distortion. The classic surgery described was a handsewn anastomosis. Lately a stapled approach has emerged which is less technically demanding, and requires shorter operative time.

We compared stapled versus handsewn Kono-S ileocolonic anastomosis in patients with Crohn’s disease, evaluating operative time and perioperative outcomes.

**Methods:**

Data on all consecutive patients with CD aged ≥ 18 years at a single tertiary center, who underwent ileocolonic resection by inflammatory bowel disease (IBD)-dedicated surgeons with Kono-S anastomosis from July 2023 to April 2025, were collected retrospectively.

**Results:**

In total, 25 patients were included. Overall, 15 (60%) underwent handsewn anastomosis and 10 (40%) underwent stapled anastomosis. There were no clinical or demographic differences. Median operative time was shorter in the stapled group (151 versus 203 min, *p* = 0.01). Postoperative complications occurred in 2/10 patients (20%) in the stapled group and 4/15 (26.7%) in the handsewn group (*p* = 0.70). One patient required reoperation in the handsewn group. Postoperative day 3 C-reactive protein (CRP) was lower in the stapled group (median 69 versus 165 mg/L, *p* = 0.03). There was one case of 30-day rehospitalization in the stapled group.

**Conclusions:**

The stapled Kono-S anastomosis technique is a shorter procedure with similar perioperative outcomes compared with the handsewn technique. Follow-up studies, with larger sample sizes, are required to evaluate long-term efficacy and disease recurrence rates.

## Background

Crohn's disease (CD), a chronic inflammatory disease that can affect the entire gastrointestinal tract, is managed by both medical and surgical means. In recent years medical treatment has made significant advancement, yet, as many as 50% of patients with CD require surgery within 10 years of diagnosis [1, 2]. The most common location of inflammation is the distal ileum, and the most common surgery for CD is ileocecal resection (ICR). Postoperative recurrence (POR) is common, as nearly 50% of patients have clinical recurrence and about 30% will eventually require another resection [3]. As most recurrences occur in the anastomosis, it has been debated whether surgical technique has an impact in reducing POR [4, 5].

In 2003, antimesenteric handsewn functional end-to-end anastomosis Kono-S, designed to minimize anastomotic restenosis, was first performed in Asahikawa Medical University Hospital by Kono Toru et al. [6, 7]. This technique has been shown to reduce the incidence of endoscopic anastomotic recurrence and need for reoperations compared with conventional side-to-side anastomosis [8, 9]. The downside of this technique has been the longer operation time and the complexity of the procedure. Lately, a stapled approach has emerged, suggested to be less technically demanding and requiring shorter operative times [10, 11]. However, no study has directly compared the handsewn and stapled techniques.

In this study, we compared operative time and perioperative outcomes of stapled versus handsewn Kono-S ileocolonic anastomosis in patients with CD.

## Methods

**Fig. 1:**
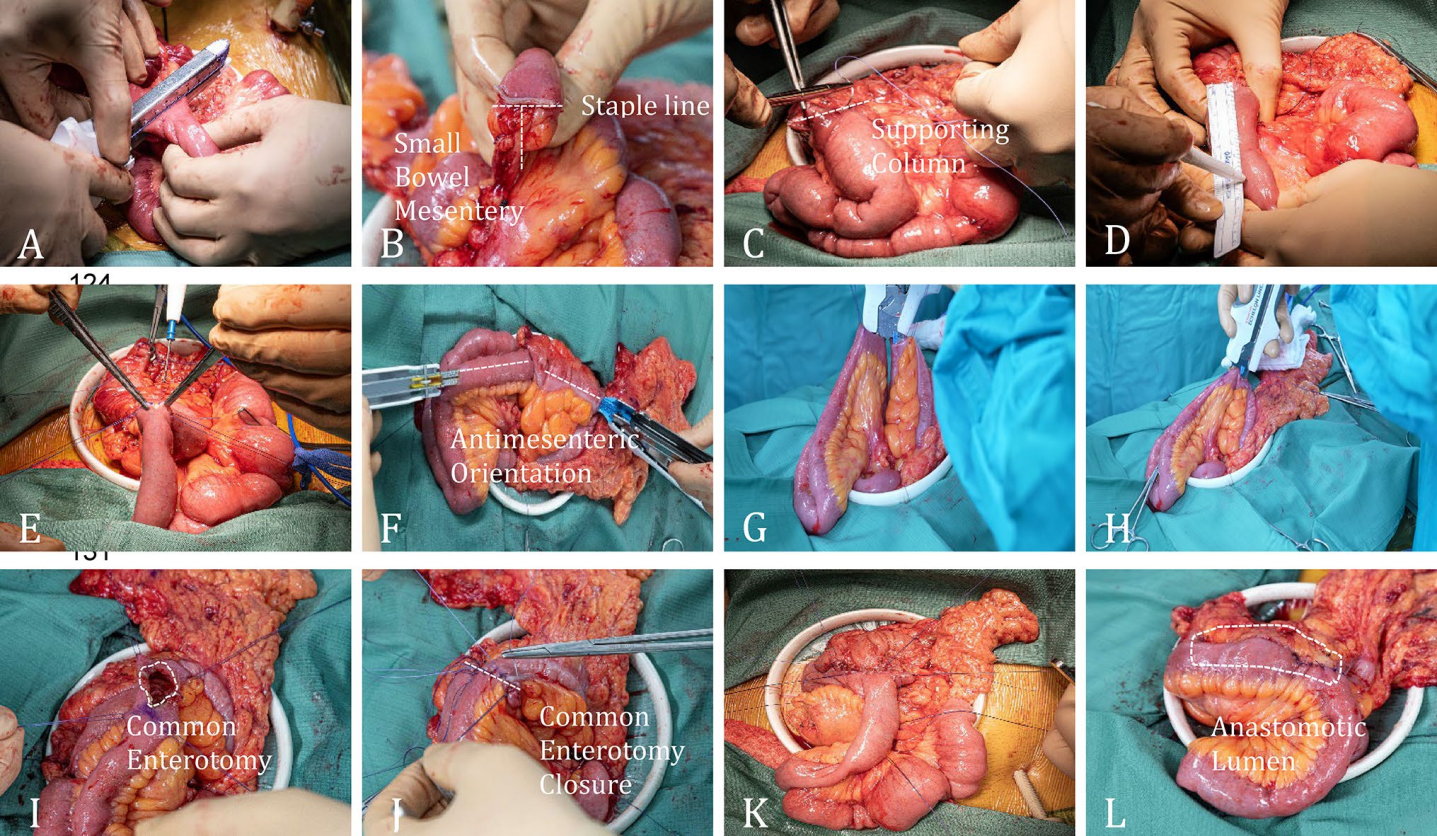
Stapled Kono-S technique. **A** Small bowel (SB) resection. **B** Stapler line perpendicular to the mesentery. **C** Creating a posterior suture line as a supporting column for the anastomosis. **D** Measuring 8 cm distance for the enterotomy. **E** SB enterotomy. **F** Inserting linear stapler to the SB and colon. **G** Approximating the limbs of the anastomosis with holding sutures. **H** Creating a side-to-side functional end-to-end antimesenteric anastomosis (Kono-S). **I** The common enterotomy. **J** Continuous handsewn closure of the common enterotomy. **K** Closure of common enterotomy. **L** Final anastomosis

This was a single-center, observational cohort study. The study included all consecutive patients with a confirmed diagnosis of CD, who underwent an antimesenteric functional end-to-end Kono-S anastomosis for restoring bowel continuity after ICR at the Tel Aviv Medical Center from 11 July 2023 to 3 April 2025. Only handsewn and stapled Kono-S anastomoses were included, while other types of anastomosis were excluded. The handsewn anastomosis was done as described in previous studies [6], whereas the stapled Kono-S anastomosis was done with the same principles while using a GIA™ or ECHELON Linear Cutter™ with 80 mm length, 3.8/1.5 mm (open/close) staples and 3.7/1.5 mm (open/close), respectively. The common enterotomy was closed perpendicularly to the anastomosis direction and in continuation with the staple line. Second layer was done with interrupted 3–0 Vicryl (Fig. 1). The decision to perform stapled Kono-S anastomosis relied on temporal evolution, as after experiencing the meticulous, highly demanding handsewn anastomosis, we sought an easier, reproducible, and safe technique, and started performing stapled Kono-S anastomoses.

Baseline demographic and clinical characteristics included age, gender, body mass index (BMI), smoking history, disease duration, Montreal classification, previous intestinal resections, medical treatment, and indication for surgery. Intraoperative details including surgical approach (laparoscopic, laparotomy, or conversion), anastomosis type, and operative time were also collected. In addition, postoperative outcomes including length of stay, complications (leak, abscess, ileus, bleeding), postoperative C-reactive protein (CRP), Clavien–Dindo score, and 30-day readmission rates were evaluated. Finally, for those available, postoperative endoscopic findings, including Rutgeerts scores, were recorded.

The primary outcome was postoperative complications according to the Clavien–Dindo classification [12]. Other secondary outcomes included length of operation, CRP on postoperative day 3, length of stay, and 30-day readmission.

### Statistical analysis

Continuous variables are presented as means ± SD or medians (IQR) on the basis of normality distributions assessed using the Shapiro–Wilk test. Categorical variables were described as frequencies and percentages. The Student’s *t* test or Mann–Whitney *U* test was used to compare the differences between groups for continuous variables, depending on the normality of the data. The Pearson chi-squared test or Fisher’s exact test was used to compare categorical variables, as appropriate. Statistical analyses were performed using the IBM SPSS software (version 29.0; IBM). A two-sided *p* value of < 0.05 was regarded as statistically significant.

## Results

### Baseline characteristics

During the study period, 25 patients underwent ileocolic resection with an antimesenteric functional end-to-end (Kono-S) anastomosis. In total, 10 patients (40%) had a stapled Kono-S anastomosis and 15 (60%) had a handsewn Kono-S. Baseline demographic and disease-related variables were comparable, as shown in Table 1. Median age was 35 years (IQR 28–41 years) in the stapled group and 36 years (IQR 25–64 years) in the handsewn group (*p* = 0.50). Median disease duration, BMI, smoking status, Montreal behavior, ASA class, previous resections, and surgical indication did not differ significantly. There were no differences in terms of biological treatments prior to surgery (seven patients in the stapled group and nine in the handsewn group). However, there was a significant difference in the type of biological treatment received; anti-interleukin (anti-IL)12/23 was more common in the stapled group versus anti-tumor necrosis factor (anti-TNF) being more common in the handsewn group.

**Table 1:** Baseline characteristics

	Stapled Kono-S (*n* = 10)	Handsewn Kono-S (*n* = 15)	*p*-Value †
Demographics			
Age at surgery, years—median (IQR)	35 (28–41)	36 (25–64)	0.50
Sex, female—no. (%)	7 (70%)	7 (53.3%)	0.25
Body mass index, kg/m^2^—median (IQR)	22.3 (20.5–26.2)	22.7 (20.7–25.7)	0.86
Smoking—no. (%)			0.20
Active	3 (30%)	3 (20%)	
Past	0 (0%)	4 (26.7%)	
No	7 (70%)	8 (53.3%)	
Disease Characteristics			
Crohn’s disease duration, years—median (IQR)	12 (5–23)	7 (3–25)	0.64
Montreal classification behavior—no. (%)			0.12
Stricturing	7 (70%)	14 (93.3%)	
Penetrating	3 (30%)	1 (6.7%)	
ASA score II—no. (%)	10(100%)	15 (100%)	
Previous surgeries—no. (%)	1 (10%)	5 (33.4%)	0.18
Biological treatment—no. (%)	7 (70%)	9 (60%)	0.60
Anti-IL12/23	7 (100%)	2 (22.2%)	0.08
Anti-integrin	0 (0%)	1 (11.1%)	
Anti-TNF	0 (0%)	6 (66.7%)	
Steroid treatment—no. (%)	1 (10%)	2 (13.3%)	0.80
Indication for surgery—no. (%)			0.76
Stricture	9 (90%)	14 (93.3%)	
Fistula/abscess	1 (10%)	1 (6.7%)	

^†^Continuous variables compared with Student’s *t*-test (normal distribution) or Mann–Whitney *U* test (nonparametric); categorical variables compared with *χ*^2^ or Fisher’s exact test (two-sided)

### Operative characteristics

**Fig. 2:**
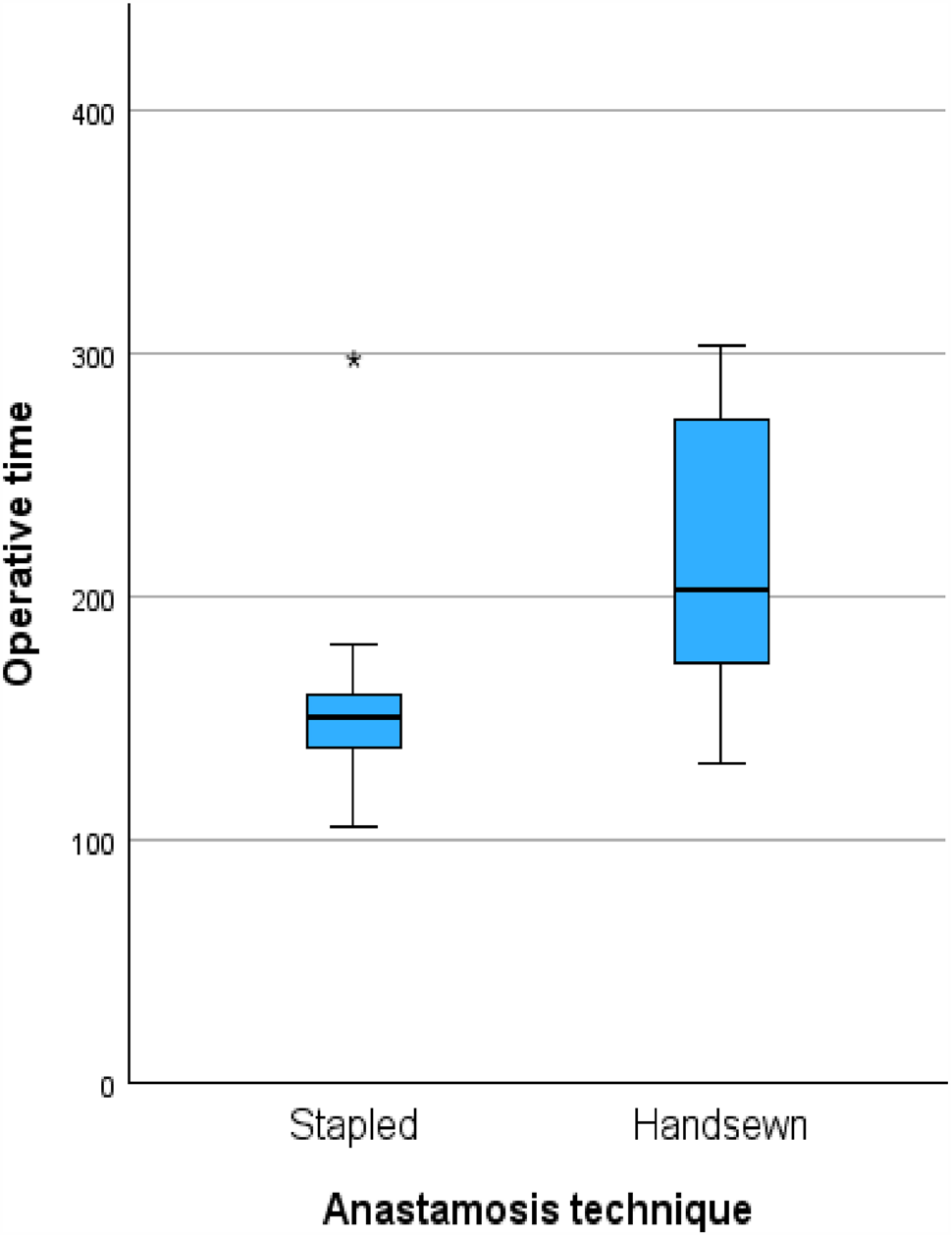
Operative time (min) compared between stapled and handsewn anastomosis

Operative details as summarized in Table 2 show that operative time was shorter in the stapled group (151 versus 203 min, median difference 52 min, 95% CI 4–100 min, *p* = 0.01) (Fig. 2). None of the patients in the stapled group had a concomitant bowel resection, while in the handsewn group, one patient had a gastrojejunostomy due to duodenal stricture. Even after excluding this case the operative time remained significantly shorter in the stapled group. Estimated blood loss was uniformly low and no patient in either cohort required intra-operative blood transfusion. Two patients in the handsewn group required conversion from laparoscopy to open surgery due to difficulty in mobilizing the colon. In order to avoid bias due to case complexity, after excluding these two cases and performing subgroup analysis, operative time was still statistically significantly shorter in the stapled group (151 versus 203 min, median difference 52 min, *p* = 0.02).

**Table 2:** Intra-operative details

	Stapled Kono-S (*n* = 10)	Hand-sewn Kono-S (*n* = 15)	*p*-Value †
Intra-operative details			
Operative time, min—median (IQR)	151 (133.5–165.3)	203 (173–282)	0.01
Laparoscopic conversion to open—no. (%)	0 (0%)	2 (13.3%)	0.20
Concomitant bowel resection—no. (%)	0 (0%)	1 (6.7%)	0.40
Estimated blood loss (> 100 mL)—no. (%)	0 (0%)	0 (0%)	
Intra-operative blood transfusion—no. (%)	0 (0%)	0 (0%)	

^†^ Continuous variables compared with Student’s *t*-test (normal distribution) or Mann–Whitney *U* test (nonparametric); categorical variables compared with *χ*^2^ or Fisher’s exact test (two-sided)

### Postoperative outcomes

**Fig. 3:**
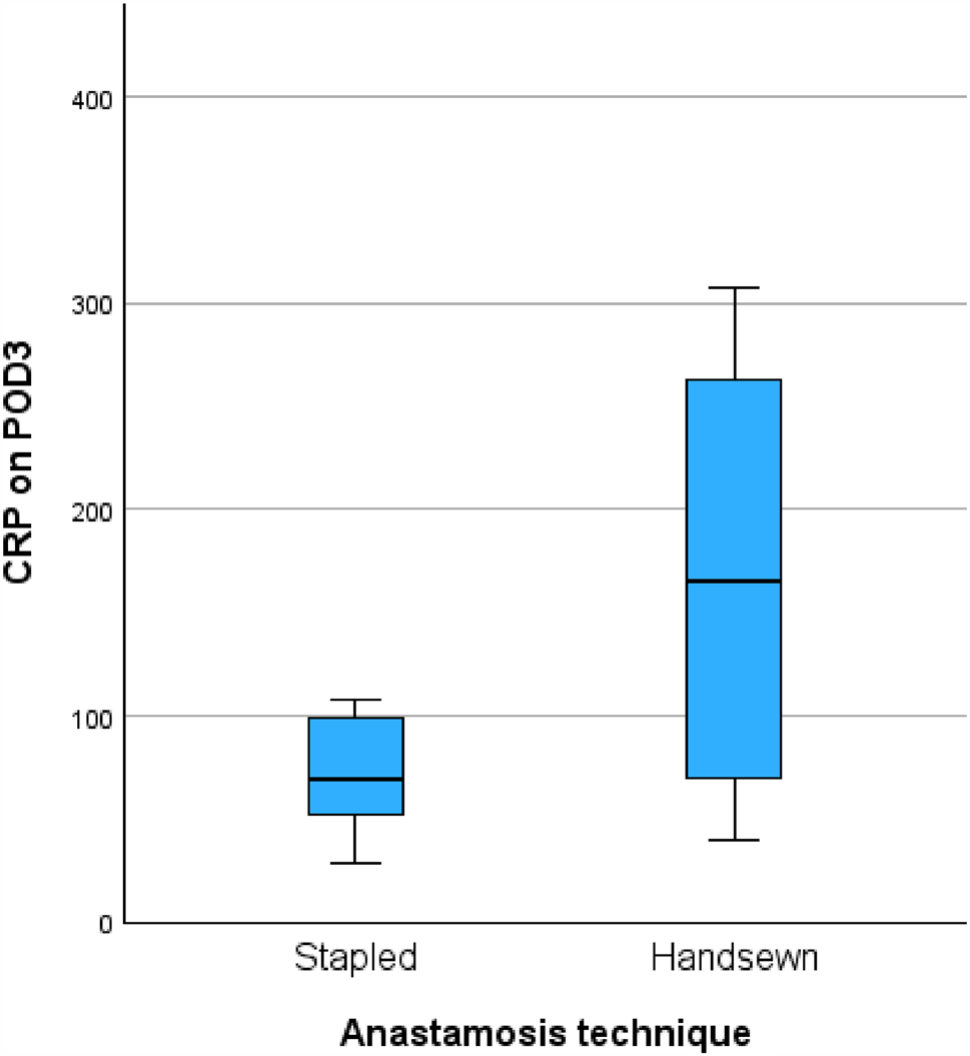
CRP (mg/L) on postoperative day 3 compared between stapled and handsewn anastomoses

Overall 30-day morbidity was similar between techniques (Table 3). Postoperative day 3 CRP was lower in the stapled group (median 69 versus 165 mg/L, a difference of 96 mg/L, 95% CI 17–175 mg/L, *p* = 0.029) (Fig. 3). Minor complications, Clavien–Dindo score ≤ II, occurred in 40% of the stapled group patients and 20% of handsewn group patients (*p* = 0.28). Anastomotic leak was presumed according to computed tomography (CT) scan in two patients of the stapled group (20%) and two patients of the handsewn group (13%) (*p* = 0.65). All four patients were treated with antibiotics, and one of these patients in the handsewn group required reoperation and reinforcement of the anastomosis with temporary ileostomy. Accordingly, major complications (Clavien–Dindo score ≥ III) occurred only in the aforementioned patient, and no statistical significance was seen. Postoperative ileus occurred in one patient in the stapled group and none in the handsewn group (*p* = 0.21). Postoperative intraluminal hemorrhage was recorded in two patients in the stapled group, none requiring transfusion, and one patient in the handsewn group (*p* = 0.32), who had a 4 g/dL drop in hemoglobin and required two packed cell transfusions.

**Table 3:** Early postoperative outcomes (≤ 30-day)

	Stapled Kono-S (*n* = 10)	Handsewn Kono-S (*n* = 15)	*p*-Value †
Early postoperative outcomes (≤ 30-day)			
CRP on postoperative day 3, mg/L—median (IQR)	69 (46–101.3)	165 (68–267)	0.03
Minor complication (Clavien–Dindo ≤ II)—no. (%)	4 (40%)	3 (20%)	0.28
Major complication (Clavien–Dindo III–V)—no. (%)	0 (0%)	1 (6.7%)	0.40
Anastomotic leak—no. (%)	2 (20%)	2 (13.3%)	0.65
Postoperative ileus—no. (%)	1 (10%)	0 (0%)	0.21
Postoperative hemorrhage—no. (%)	2 (20%)	1 (6.7%)	0.31
Re-operation within 30 days—no. (%)	0 (0%)	1 (6.7%)	0.40
Readmission within 30 days—no. (%)	1 (10%)	0 (0%)	0.21
Length of hospital stay, days—median (IQR)	5 (5–10)	7 (6–8)	0.26
30-day mortality—no. (%)	0 (0%)	0 (0%)	

^†^Continuous variables compared with Student’s *t*-test (normal distribution) or Mann–Whitney *U* test (nonparametric); categorical variables compared with *χ*^2^ or Fisher’s exact test (two-sided)

Median length of hospital stay was 5 days (IQR 5–10 days) for the stapled group and 7 days (IQR 6–8 days) for the handsewn group (*p* = 0.26). One patient in the stapled group was readmitted within 30 days due to intraluminal hemorrhage, however, no intervention was required. There was no 30-day mortality in either group.

## Discussion

This single-center series shows that stapled Kono-S anastomosis compared with the handsewn technique significantly reduced operative time by a median of 52 min (151 versus 203 min). While achieving this advantage, no difference was seen in the 30-day outcomes, including anastomotic leak, Clavien–Dindo complications, re-operation, length of stay, or readmission rate, which were not meaningfully different from the traditional handsewn technique. Interestingly, the CRP levels on postoperative day 3 were significantly lower in the stapled group.

Our 25% reduction in operation time is similar to the reduction reported in previous single-center comparisons of non-Kono-S stapled versus handsewn intestinal anastomoses in CD [11]. Meta-analysis data focusing on Kono-S showed no overall difference in mean operative time versus conventional side-to-side stapling, but heterogeneity was high and few studies isolated the stapled Kono-S variant [13]. The present cohort, therefore, provides the first evidence, controlling for both medical center and operating surgeons, that stapling specifically accelerates the Kono-S construction without compromising early postoperative safety.

Anastomotic leak, ileus, and re-operation rates in a previous systematic review of the Kono-S technique, report a leak rate of around 1–5% and major morbidity (Clavien–Dindo > 3) < 10% [14]. Although the absolute leak rate in our study was higher, the major morbidity rate was similar and might be attributed to the small sample size. The absence of a between-group difference compared with larger CD cohorts comparing standard side-to-side (non-Kono-S) stapled anastomoses with handsewn anastomoses [11] suggests that switching from handsewn to staples does not compromise safety. A possible explanation of the reportedly high leak rate might be due to the wide variety of criteria used in our unit for anastomotic leakage reporting, as mentioned above, only one patient had a surgically proven leak in the handsewn group; therefore, it might lower the confirmed leak rate to 6% (1/15), which is similar to other studies [14].

In addition, CRP on postoperative day 3 is a validated leak surrogate after right-sided colectomy, with alert thresholds ranging from 118 to 166 mg/L [15, 16], and while our stapled group median postoperative day 3 CRP was well below this range, the handsewn median exceeded the upper threshold, suggesting additional safety potential of stapled anastomoses.

Our study has several limitations. First, its small sample size restricts generalizability and statistical power. While our findings regarding operative time and early postoperative outcomes are notable, the study is not powered to detect small differences in low-incidence events such as complications or reoperation. A post hoc calculation based on 80% power and *α* = 0.05 showed that detecting the observed 50-min difference in operative time would require approximately 23 patients per group. Detecting a 20% absolute difference in complication rates would require around 90 patients per group. These estimates emphasize the exploratory nature of our findings, which correspond to a stage 2a investigation within the IDEAL framework for surgical innovation as an early, single-center, developmental study designed to assess the feasibility and short-term outcomes of a novel adaptation of the Kono-S anastomosis. As such, our results should be interpreted as hypothesis-generating and intended to inform future, prospective studies. Second, the short follow-up time limits our ability to show whether the stapled anastomosis maintains the reduction in endoscopic and surgical recurrence shown in the handsewn technique, as previously reported, thus requiring further follow-up and consecutive study.

Our strengths include the consecutive enrollment in a single center with a uniform surgical technique and peri-operative pathway, objective leak criteria, and systematic CRP monitoring. Furthermore, while previous studies and current recruiting studies, such as HAND2END and End2End [17], have compared different techniques, our study is the first to directly compare stapled versus handsewn Kono-S anastomosis. Looking ahead, integration into IBD surgical registries and collaborative prospective data sharing will be essential for evaluating the generalizability, reproducibility, and long-term outcomes of the stapled Kono-S technique. Such registries can play a critical role in standardizing reporting, enabling multi-institutional comparisons, and guiding evidence-based adoption of novel surgical approaches.

## Conclusions

Converting the functional end-to-end ileocolic anastomosis Kono-S from handsewn to a stapled technique significantly reduces the operative time while lowering postoperative CRP levels and without compromising 30-day peri-operative outcomes. We believe that this allows standardization of the technique and shows feasibility and safety of the procedure. Further studies are needed to show advantages in endoscopic, clinical, and surgical recurrences.

## Data Availability

The data that support the findings of this study are not openly available due to reasons of sensitivity and are available from the corresponding author upon reasonable request. Data are located in controlled access data storage at Tel Aviv Sourasky Medical Center.
